# VASP-E: Specificity Annotation with a Volumetric Analysis of Electrostatic Isopotentials

**DOI:** 10.1371/journal.pcbi.1003792

**Published:** 2014-08-28

**Authors:** Brian Y. Chen

**Affiliations:** Department of Computer Science and Engineering, P.C. Rossin College of Engineering and Applied Sciences, Lehigh University, Bethlehem, Pennsylvania, United States of America; UNC Charlotte, United States of America

## Abstract

Algorithms for comparing protein structure are frequently used for function annotation. By searching for subtle similarities among very different proteins, these algorithms can identify remote homologs with similar biological functions. In contrast, few comparison algorithms focus on specificity annotation, where the identification of subtle differences among very similar proteins can assist in finding small structural variations that create differences in binding specificity. Few specificity annotation methods consider electrostatic fields, which play a critical role in molecular recognition. To fill this gap, this paper describes VASP-E (Volumetric Analysis of Surface Properties with Electrostatics), a novel volumetric comparison tool based on the electrostatic comparison of protein-ligand and protein-protein binding sites. VASP-E exploits the central observation that three dimensional solids can be used to fully represent and compare both electrostatic isopotentials and molecular surfaces. With this integrated representation, VASP-E is able to dissect the electrostatic environments of protein-ligand and protein-protein binding interfaces, identifying individual amino acids that have an electrostatic influence on binding specificity. VASP-E was used to examine a nonredundant subset of the serine and cysteine proteases as well as the barnase-barstar and Rap1a-raf complexes. Based on amino acids established by various experimental studies to have an electrostatic influence on binding specificity, VASP-E identified electrostatically influential amino acids with 100% precision and 83.3% recall. We also show that VASP-E can accurately classify closely related ligand binding cavities into groups with different binding preferences. These results suggest that VASP-E should prove a useful tool for the characterization of specific binding and the engineering of binding preferences in proteins.

## Author Summary

Proteins, the ubiquitous worker molecules of the cell, are a diverse class of molecules that perform very specific tasks. Understanding how proteins achieve specificity is a critical step towards understanding biological systems and a key prerequisite for rationally engineering new proteins. To examine electrostatic influences on specificity in proteins, this paper presents VASP-E, a software tool that generates solid representations of the electrostatic potential fields that surround proteins. VASP-E compares solids with constructive solid geometry, a class of techniques developed first for modeling complex machine parts. We observed that solid representations could quantify the degree of charge complementarity in protein-protein interactions and identify key residues that strengthen or weaken them. VASP-E correctly identified amino acids with established experimental influences on protein-protein binding specificity. We also observed that solid representations of electrostatic fields could identify electrostatic conservations and variations that relate to similarities and differences in binding specificity between proteins and small molecules.

This is a *PLOS Computational Biology* Methods article.

## Introduction

Software for comparing protein structures is widely used to make inferences about protein function. These methods assist in function annotation by revealing proteins that perform similar biological functions despite vast evolutionary differences. Many methods focus on the discovery of subtle structural similarities among very different molecules [1], [2] using the superposition of catalytic residues [3]–[5] or the comparison of binding cavities [6]–[9]. By aligning polypeptide backbones [10]–[14], distance matrices [15] or geometric graphs [16]–[18], related methods can reveal similarities in tertiary structure that are not evident from sequences alone. Most approaches use atom coordinates or molecular surfaces [19]–[22] as digital representations of protein geometry. Other characteristics, such as evolutionary significance [23]–[25], hydrophobicity [26] and electrostatic potential [5], [23], [27] are attached to this geometric representation as labels. Comparisons of these data often generate a score, such as the root mean squared distance (RMSD), that summarizes structural, biological, and chemical similarities among two or more structures. Proteins with very different sequences sometimes exhibit unusually similar RMSDs, revealing shared origins in antiquity [28]–[30].

An emerging second type of comparison algorithm is designed to find subtle differences among very similar proteins. These methods seek to annotate protein specificity by proposing structural causes for different binding preferences among proteins that perform the same function [31]–[36]. For example, specificity annotation software might identify a cleft inside the ligand binding cavity of one protein that does not exist in a close homolog. That cleft might accommodate substrates that the homolog cannot bind. To find structural features like these, RMSD, and other scores for function annotation, are inappropriate because they obscure meaningful individual variations, like the cleft, behind summary scores. Instead, volumetric representations [37], which describe protein structure based on spatial regions occupied by the atoms of a protein, can be used to identify individual structural differences that may alter the binding preferences of ligand binding cavities [31]–[34]. To date, few comparisons focused on finding subtle electrostatic differences among closely related proteins have been reported, even though electrostatic fields are widely used to infer protein function [38]–[47] and specificity [48]–[53]. To fill this gap, this paper proposes a novel volumetric representation and comparison algorithm for finding electrostatic influences on binding specificity.

**Figure 1:**
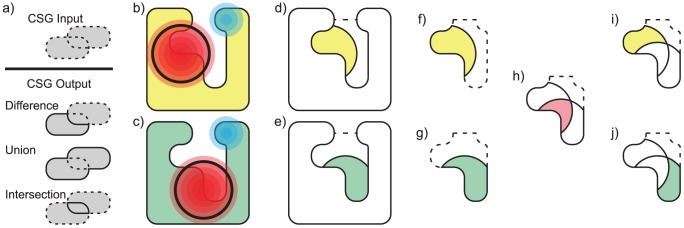
CSG analysis of electrostatic isopotentials in ligand binding cavities. a) A demonstration of CSG operations, illustrating the borders of input (dotted) and output (solid) regions in grey (grey everywhere). b,c) Shapes representing the regions occupied by protein **X** (yellow) and **Y** (green), their molecular surfaces (thin black lines), and their electrostatic potential fields (red and blue gradients). Regions with increasingly negative potential are shown in darker red, and regions with increasingly positive potential are shown in darker blue. An isopotential selected by a user is shown with a heavy black line. d,e) The CSG differences **x** and **y** between the region within the user-selected isopotential and the molecular surface of **X** and **Y** is shown in yellow and green. These volumes represent solvent accessible cavity regions with electrostatic potential at least as negative as that selected by the user. The external boundary of the ligand binding cavities of **X** and **Y** is shown with a dotted line. f,g) **x** and **y** are shown in yellow and green, with a black boundary. The ligand binding cavities they occupy are shown with a dotted boundary. h) The CSG intersection (red) of **x** and **y** (black outlines), when **X** and **Y** are aligned, represents a solvent accessible cavity region where electrostatic potential in both proteins is at least as negative as that selected by the user. i,j) The CSG differences of **x** - **y** (yellow) and **y** - **x** (green), indicating regions of electrostatic potential in the ligand binding cavity of one protein and not the other.

The problem we are specifically addressing is the case where several closely related proteins have already been structurally aligned and we seek to identify spatially conserved and varying regions in their potential fields that might cause differences in binding specificity. Conserved regions, where the fields have similar potentials, might stabilize a molecular fragment attracted by all proteins (Fig. 1g), while differences in specificity could arise from regions where the fields vary (Fig. 1h,i). Software for identifying conservation and variation in charged regions can thus suggest how such regions may play a role in molecular recognition, and how they might be changed to achieve different binding preferences. Our approach identifies regions like these by representing electrostatic isopotentials with volumetric solids generated by the new program VASP-E (Volumetric Analysis of Surface Properties with Electrostatics). VASP-E computes conserved and varying regions using techniques from Constructive Solid Geometry (CSG) (Fig. 1). Developed originally for computer aided design [54] and computer graphics [55], CSG enables unions, intersections, and differences of volumetric representations to be calculated as if they are three dimensional solids. When used to analyze fields, CSG intersections can approximate regions that are common to isopotentials from several aligned proteins, thereby identifying regions with conserved potentials. CSG differences identify regions inside the isopotential of one protein but not inside that of another, isolating a region where potentials vary. Together, CSG operations provide a novel mechanistic approach to the analysis of electrostatic fields because the approximation of conserved and varying fields is not possible with existing structure comparison methods.

The solid representations employed by VASP-E differ in kind from existing electrostatic analyses. While VASP-E deconstructs the electrostatic field to identify conserved and varying electrostatic phenomena, existing methods summarize and quantify the field with comparison scores [56], [57] and biophysical energies [58]–[61]. These numerical values cannot point to specific regions in the field with electrostatic similarities or differences, and so they cannot suggest how a protein might be altered to engineer different binding preferences. A second fundamental difference is that solid representations have the additional capability to represent the region inside molecular surfaces. Using CSG, we can therefore integrate both types of data to focus on electrostatic fields within binding sites. For example, the CSG difference of an isopotential minus the molecular surface at a binding site represents a three dimensional charged region in the solvent that can be occupied by potential binding partners (e.g. Fig. 1c,d). In contrast, representations used in function annotation methods generally represent electrostatic fields at or near the molecular surface only. Sampling a three dimensional field along this curving two dimensional surface cannot describe the electrostatic field as it extends outwards from the protein and influences other molecules. Third, while existing methods characterize fields at all potentials, solid representations describe fields at selected isopotential thresholds only. This feature enables comparisons to focus on ranges of potential that are especially relevant to binding. It can also be used to measure electrostatic complementarity between binding partners, as we will demonstrate later, by identifying interface regions where oppositely charged isopotentials overlap. To our knowledge, VASP-E is the first application of CSG to the volumetric comparison of electrostatic isopotentials, although tree-based methods that summarize topological differences in electrostatic isopotentials [57] have also been developed.

This paper explores two applications of VASP-E as it might be applied in support of research in structural biology. One objective in many investigations is to discover electrostatic influences on protein-ligand or protein-protein binding specificity. Given the long range nature of electrostatic interactions, many amino acids could potentially be influential, and it could be impractical to create all possible mutants and determine their binding preferences. Here, a first application of VASP-E is to suggest amino acids that create differences between the electrostatic fields of two ligand binding cavities or to suggest amino acids that enhance or diminish electrostatic complementarity between two interacting proteins. Because amino acids are suggested in tandem with a hypothetical electrostatic influence on binding, VASP-E provides reasons to produce and test certain mutants first, where no reason might have existed before. The second application of VASP-E examined in this paper is the classification of protein-ligand binding cavities based on their electrostatic fields. This application can support efforts to discover patterns of electrostatic similarities or differences among related binding sites. In studies seeking to identify a possible ligand, electrostatic classification can reveal similarities to other proteins that may have known binding partners. Together, these applications of VASP-E represent two of many capabilities that become possible by combining CSG and volumetric representations of electrostatic isopotentials. We validate these capabilities in the results section against established experimental observations.

## Methods

### 2.0.1 Method summary

The underlying observation exploited by VASP-E is that geometric comparisons of electrostatic potential fields can focus on biologically relevant regions and specific potential ranges by using CSG. Constraining the comparison of potential fields in this manner ensures that comparisons reflect aspects of electrostatic fields that influence binding, rather than spurious variations that occur by random chance or outside of binding sites. To achieve this kind of focus, comparisons always begin with a multiple structure alignment of whole proteins [10]–[18], [62], where ligand binding cavities or protein-protein interfaces are defined on each structure using cavity detection algorithms [63]–[67] or manual design.

Structures aligned in this manner are then used to generate solid representations of electrostatic isopotentials and protein structure. To represent electrostatic isopotentials, we first solve the potential field of a given structure using DelPhi [68]. Using the field, isopotential surfaces are approximated using Marching Cubes [69], an algorithm first applied to visualize electrostatic isopotentials in GRASP [70]. This method is paraphrased below. Solids representing molecular surfaces are generated using the Trollbase library [12], which implements the classical rolling-probe method [71].

The resulting solids, regardless of their origin, are basic inputs for CSG operations, which we described earlier [37]. Below, we use the symbols 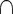, 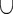 and 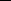 to denote intersection, union, and difference operations, which are the basic CSG operations used in this work. VASP-E uses CSG to integrate solid representations of electrostatic isopotentials and molecular surfaces to create solid representations of the electrostatic field within ligand binding cavities (*cavity fields*) and protein-protein interfaces (*interface fields*). These procedures are detailed below.

Cavity fields and interface fields are the constrained representations used by VASP-E to focus the comparison of electrostatic fields on biologically significant regions. To quantify similarities, we compute the CSG intersection of two regions and then evaluate the volume of the resulting intersection region. To quantify differences, we measure the volume of the CSG difference. Large volumes of intersection imply similar fields while large differences are characteristic of fields that vary. To estimate the volume 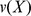 of any region 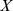, including outputs from CSG operations, we use the Surveyor's Formula [72], which we described earlier [37].

Further CSG operations permit deconstructive comparisons of cavity and interface fields that identify similarities in some regions and differences in other regions within the fields they describe. While many applications this kind are possible with VASP-E, we describe two below: First, we can use VASP-E to trace differences in electrostatic fields to individual amino acids that contribute to these differences, thereby predicting residues that influence binding specificity. Second, we can integrate multiple electrostatic similarity measurements between a family of cavity fields to reveal patterns of ligand binding specificity.

### 2.1 Solid representations of electrostatic isopotentials with marching cubes

**Figure 2:**
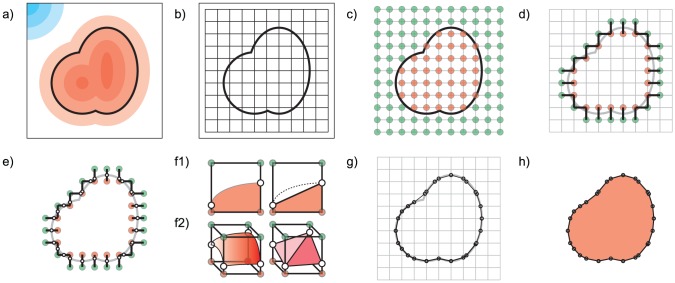
Generating a solid representation of an electrostatic isopotential using marching cubes. a) The input electrostatic field, illustrated as a gradient of red (negative potential) and blue (positive potential) regions. The solid region to be approximated is within the heavy black line. b) Axis aligned cubic lattice surrounding solid isopotential (black grid). c) Lattice points (circles) evaluated as being inside (red) or outside (green) the isopotential. d) Selected edges, found between interior and exterior lattice points (short black lines), intersect the electrostatic isopotential (grey curved line). e) Intersection points along each selected edge (small white circles). f1) A two dimensional illustration of the solid isopotential passing through a lattice square (red, left), with interior lattice points shown with red circles, and exterior lattice points shown with green circles. An approximation of the solid isopotential using a straight line is shown on the right. f2) A three dimensional illustration of the surface of a solid isopotential (red gradient, left) inside a lattice cube. Lattice points inside the solid isopotential are shown as red circles, lattice points outside are shown in green. An approximation of the solid isopotential triangles connecting intersection points (white circles) is shown on the right. g) Together, the triangles in all cubes (black lines) form the boundary surface approximating the solid isopotential (h).

As input, marching cubes begins with a molecular structure from the Protein Data Bank (PDB) [73], its electrostatic potential field 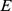, a desired isopotential threshold 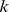, and the user's choice of representing the region with potential greater than or less than 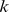. The overall procedure (Fig. 2) approximates the solid region on one side of the isopotential at 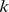, which we refer to as a *solid isopotential* (Fig. 2a). In this work, when generating electrostatic isopotentials at 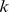 kT/e, we always represent the region with potential greater than 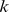 when 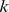 is positive, and the region with potential less than 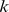, when 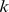 is negative. Regions on the other sides of these potentials are infinite in volume, and thus their comparison is not well defined. Below, we use a negative value for 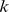 and represent the region on the lower-potential side of 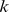, as an example.

First, we protonate the PDB structure using the *reduce* component of MolProbity [74]. The resulting structure is passed to DelPhi [68], which computes numerical solutions to the nonlinear Poisson-Boltzmann equation, yielding an approximation of 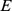 at every point within a bounding box surrounding the protein. Using 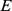, Marching Cubes outputs a polyhedral approximation of the isopotential surface at k kT/e, which we interpret as the exterior boundary of a three dimensional solid.

Marching Cubes begins by establishing a regular lattice of cubes around the protein, whose borders fall within the bounding box (Fig. 2b). The lattice as a whole can be interpreted as a collection of *lattice points* at the corners of each cube, *lattice edges* connecting adjacent corners, *lattice faces* between cubes, or as simply a collection of *lattice cubes*. The *resolution* of the lattice, defined by the length of a lattice edge, is specified by the user and can be changed to accommodate structures of different sizes in system memory.

Once the lattice is initialized, we evaluate the potential 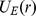 of the field 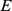 at every lattice point 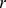. If 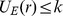, we mark 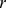 as being *inside* the isopotential. Otherwise, we mark 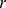 as being *outside* (Fig. 2c). The evaluation of 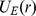 is made possible using the Trollbase library [12], which evaluates the field to determine the potential at 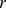.

Next, we select every lattice edge that connects an inside lattice point to one outside. Since isopotentials are topologically closed surfaces, the selected edge must intersect the desired isopotential (Fig. 2d). On each selected edge, we estimate the *intersection point* 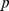 between the segment and the isopotential using linear interpolation between the electrostatic potentials at the endpoints (Fig. 2e).

Finally, we consider every lattice cube joined to at least one lattice edge with an intersection point. On the cube, the intersection points collectively approximate the places where the isopotential passes through the cube. In two dimensions, this can be drawn as a shape passing through a square (Fig. 2f1), and approximated with a line through the cube. In three dimensions, when the isopotential passes through the cube, it's boundary is a surface that intersects the segments at the intersection points calculated earlier. This surface can be approximated inside each cube with triangles connecting triplets of intersection points. We use a lookup table to specify each triangle layout based on the 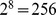 possible combinations of selected edges in a cube (Fig. 2f2). Assembling the triangles into a single surface generates the exterior boundary of the solid isopotential (Fig. 2g,h).

### 2.2 Generating and comparing cavity fields

**Figure 3:**
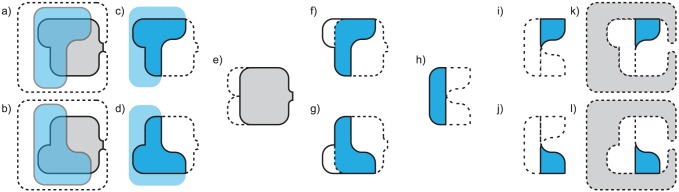
Cavity field generation and comparison. **a**,b) The molecular surfaces of protein 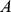 and protein 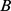 are shown in dotted outlines. Ligand binding cavities are shown in grey with solid outlines. A solid isopotential is shown in transparent blue. c,d) Cavity fields 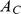 and 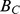 are shown in opaque blue, with the solid isopotential (transparent blue), and the binding cavity (dotted outline). e) The intersection of the two binding cavities, 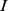 (grey) is a solvent accessible region in both cavities. f,g) The intersections 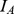 and 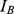 are shown in blue, 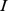 is shown with a dotted outline, and cavity fields 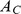 and 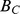 (solid outline). h) 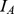 and 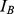 (dotted outline) and their intersection (blue). i,j) 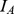 and 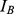 shown with a dotted outline, with differences shown in blue. k,l) The same differences relative to the molecular surface.

A cavity field is a solid representation of the region inside a ligand binding cavity that is also inside a solid isopotential. To generate a cavity field, we require the solid isopotential and a solid representation of the ligand binding cavity (Fig. 3a,b). We generate the solid isopotential using the method above and represent the ligand binding cavity using VASP and a volumetric approach based on SCREEN [65], described earlier [37]. Computing a CSG intersection of these two regions generates the cavity field (Fig. 3c,d).

We compare cavity fields to detect local electrostatic differences that might affect specificity. Our approach follows the assumption that the user has selected solid isopotentials at a threshold that is relevant for ligand binding. For example, if a negative potential is influential for the selection of positively charged substrates, comparing regions of negative potential in several cavities could reveal electrostatic causes for different binding preferences. We discuss the selection of these potentials in Supplemental Text S1.

Our comparison begins by structurally aligning two proteins, 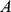 and 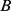, and generating their cavities, 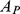 and 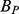. Because 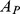 and 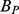 are regions that are outside the molecular surface, we say that they are *solvent accessible regions*. Using 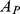 and 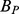, we generate their cavity fields, 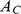 and 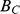 at the same side of the electrostatic potential 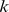. Next, we generate the intersection 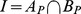. 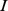 is the region that is solvent accessible in both cavities (Fig. 3e). By comparing electrostatic fields inside 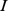, we are guaranteed that our comparison is not influenced by steric differences. For this reason, we next compute the intersection 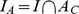 and 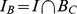 (Fig. 3f,g). 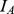 and 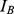 are regions within the solid isopotentials of 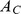 and 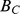 that are solvent accessible in both cavities.

Computing 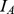 and 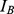 permits several useful comparisons. First, the intersection 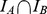 (Fig. 3h), is solvent accessible in both cavities and also inside both solid isopotentials. This region of structural and electrostatic similarity might stabilize molecular fragments that are common to substrates of both proteins. Second, the difference regions 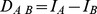 and 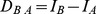 (Fig. 3i,j) are solvent accessible in both cavities but different in electrostatic character, because they lie inside the solid isopotential of one cavity and not the other. Molecular fragments in this region may thus be accommodated by one protein and electrostatically destabilized in the other.

We quantify differences by measuring the volume of 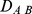 and 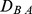. If 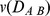 and 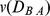 are small, we say that the cavity fields are similar. If one or both volumes are large, we say that 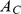 and 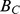 are electrostatically dissimilar, and that 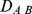 (or 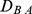) is evidence supporting the hypothesis that 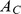 could attract or stabilize a ligand that 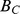 cannot. This computation enables a systematic categorization of all electrostatic differences in the binding cavities of 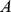 and 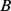.

### 2.3 Generating and comparing interface fields

An interface field is a solid representation of a region of electrostatic complementarity between two proteins 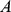 and 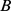, in complex. Given a potential threshold 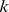, we define a region of *electrostatic complementarity* to be the spatial region where the field of 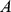, independent of 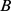, has potential greater than 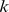, and the field of 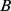, independent of 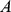, has potential less than 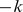. We refer to this region as an *interface field*. To generate an interface field, we require three inputs: A solid representation of the interface region, an electrostatically significant isopotential of 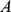 alone at 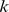 kT/e (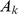), and an electrostatically significant isopotential of 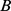 alone at 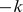 kT/e (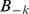). Because we use interface fields to analyze the specificity of interacting proteins, and because VASP-E is not designed to determine how two proteins interact, unbound structures are not used for the generation of interface fields.

**Figure 4:**
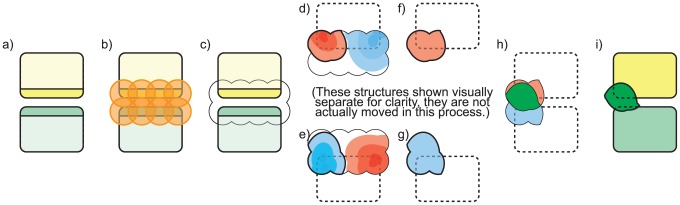
Generating an interface field. a) Two proteins in complex (rounded rectangles), and amino acids at the protein-protein interface (yellow, green). b) Spheres around every atom in the interfacial amino acids (orange). c) CSG union of interfacial spheres. d,e) Red and blue gradients representing the electrostatic potential field in the interfacial regions of protein 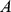 (d) and 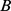 (e). Black lines represent the user-selected isopotential at 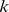 in protein 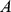 and 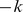 in protein 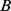. f,g) The electrostatically significant isopotentials from 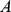 and 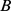, in red and blue. h) The CSG intersection (green) of the isopotentials from 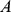 and 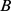. i) The interface field (transparent green).

To generate the interface region, we first identify amino acids at the interface (Fig. 4a). These are the amino acids of 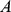 that have an atom within 5 Å of 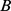, and the amino acids of 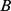 that have an atom within the same distance of 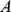. Next, we generate spheres with radius 5 Å, centered at every atom of these amino acids (Fig. 4b). Finally, we compute the interface region 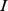 with the CSG union of these spheres (Fig. 4c). In identifying amino acids that are part of the interface region, we do not include or exclude amino acids based the fraction of their surface exposed to the solvent, because some influential “hot spot” amino acids may have low solvent exposure [75].

Electrostatically significant isopotentials 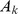 and 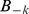 (Fig. 4d,e) are generated with the marching cubes method described above. Using 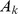 and 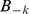, we compute 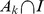, the electrostatically significant region of the field of protein 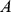 within the interface, and 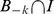, the oppositely charged electrostatically significant region of the field of protein 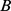 within the interface (Fig. 4f,g). The intersection of these two regions is the interface field, 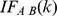 (Fig. 4h).

Since the interface field represents electrostatic complementarity in a given complex, we can use interface fields to compare electrostatic complementarity in two complexes. For two complexes, 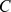 and 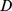, and 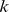, the user's threshold for electrostatic significance, we generate four interface fields: 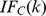, 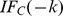, 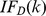, and 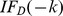. Comparing the interface fields at 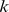 and 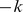 yields a more complete representation of electrostatic complementarity in both complexes.

We evaluate the difference 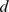 between two complexes using the following expression:
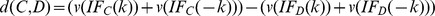



Where 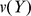 denotes the volume of a given region 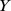. The two interface fields for each complex express the degree of complementarity on the positive and negative parts of the electrostatic potential spectrum. The interface fields for the same complex are summed, to represent the total degree of complementarity on that complex. The difference between the two sums expresses the difference in complementarity 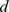 between the two complexes, on both sides of the potential spectrum. Large absolute values of 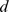 indicate large differences in electrostatic complementarity between the two complexes, while values close to zero point to similar degrees of complementarity.

### 2.4 Identifying electrostatically influential amino acids

DelPhi [68] is able to solve the electrostatic field of a given protein structure while omitting the electrostatic contribution of a individual amino acid. This process, which we refer to as *nullification*, has the unique property of leaving the structure of the amino acid intact while eliminating its electrostatic contribution. Maintaining the structure of the protein is important in an electrostatic analysis because the nullified amino acid still displaces solvent, creating a region of low dielectric. That region can enhance the electrostatic potentials of amino acids that were not nullified because of an effect called *electrostatic focusing*. Electrostatic focusing is known to play a considerable role in function and specificity [48], [76], [77]. Below, we use nullification in different ways to suggest amino acids that may influence specificity in ligand binding cavities and protein-protein interfaces. Calibration of both nullification techniques is discussed in Supplemental Text S1.

#### 2.4.1 Cavity fields

Amino acids that create electrostatic differences between two ligand binding cavities can cause different binding preferences. To identify amino acids like these, we begin with a *test* protein and a *reference* protein with different binding preferences and previously defined ligand binding cavities. First, we use ESBRI [78] to scan for intramolecular salt bridges. Second, we structurally align the test protein to the reference protein. Third, at an electrostatic threshold 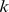 selected by the user, we compute the cavity field of the reference structure, 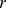, at 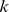. Fourth, we systematically compute variants of the electrostatic field of 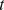, where each variant exhibits a different nullified amino acid 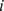. Once computed, the variant potential fields and the ligand binding cavity of the test structure are used to generate a variant cavity field 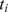, for each nullified 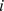, at isopotential 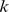.

Each nullified cavity field 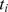 is then compared to the reference cavity field 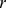 by computing the volume of their CSG difference, 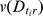. Based on this difference, we can propose several explanations for the impact of amino acid 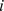 on molecular recognition: If 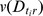 is similar to 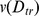, then nullifying 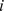 has little effect on the electrostatic differences between 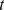 and 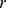, so we assume that 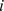 is not responsible for the differences in specificity between 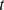 and 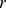. However, if 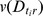 is significantly smaller than 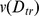, then nullification of 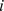 reduces electrostatic differences between 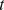 and 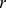. In this case, the original effect of 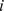 must have been to make the fields more different. Since electrostatic differences can be a sufficient reason for one protein to stabilize a binding partner that another cannot, we infer that 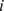 may be an electrostatic cause for different binding preferences.

Throughout this process we may observe that two amino acids 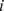 and 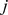 both appear, independently, to create significant differences between 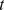 and 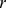. This observation, however, provides no information to compare their relative influence on specificity. Notably, even if 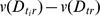 and 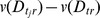 are different, they can also be differentially affected by other biophysical phenomena, and so we cannot infer that 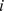 affects specificity more or less than 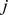 does. We may also observe that 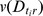 becomes greater than 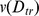, indicating that nullification of 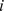 increases differences between 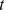 and 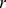, suggesting that the effect of 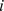 is to make the electrostatic fields of 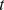 and 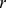 more similar. This observation may be true but it is insufficient to imply that 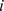 causes 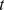 and 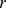 to have similar binding preferences because other biophysical differences may prevent similar molecules from binding, despite the electrostatic similarities. Finally, if we observe that an amino acid 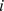 is part of an intramolecular salt bridge and that 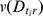 is significantly smaller than 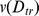, we infer that 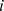 is part of a salt bridge nearby the cavity and that mutating 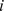 would reduce cavity stability and alter electrostatic properties inside the cavity. By evaluating every amino acid in the manner above, VASP-E yields an electrostatic analysis relating each amino acid to its possible effect on binding.

Finally, we define a conservative prediction threshold for identifying amino acids that influence specificity. First, we compute 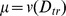, the volume of the difference between 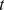 and 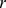 without nullifications. 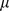 represents the baseline electrostatic differences between the two cavity fields. Second, we find the amino acid 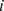 such that 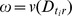 is minimized for all 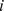. 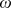 represents the maximum degree to which the nullification of an amino acid can cause 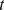 and 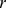 to be similar. We define the prediction threshold 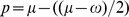, which represents electrostatic differences reduced by one half of that achieved by 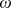. We predict that any amino acid 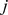, where 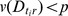, creates an electrostatic influence on specificity because nullifying it causes the cavity fields of 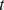 and 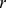 to become at least half as similar as is possible for any amino acid.

#### 2.4.2 Interface fields

For protein-protein interfaces, we can perform a similar analysis to identify amino acids that affect electrostatic complementarity. Here, we begin with the structure of an input complex 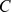 and a user-selected threshold of electrostatic potential 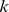. First, we use ESBRI [78] to scan for intramolecular salt bridges in each unit of the complex. We then generate interface fields at 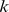 and 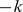. Next, we create copies of the input complex, 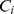, where one amino acid, 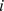, is nullified. For each variant complex 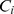, we generate interface fields at 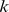 and 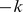 as well.

Next, we compare the interface fields of each variant complex 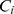 to 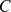, measuring the difference in electrostatic complementary 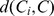 between the variant and input complexes. Using the value of the difference, we can draw several inferences about the nature of electrostatic complementarity in the complex: If 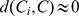 for some amino acid 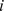, then nullifying 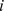 causes few differences in electrostatic complementarity at the protein-protein interface. We can thus infer that 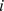 has an insignificant electrostatic influence on affinity. If for some other 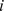, 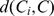 is significantly negative, then we infer that electrostatic complementarity is diminished in the variant complex relative to the input complex. 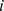 must therefore contribute to affinity when it is not nullified. Finally, if 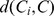 is significantly positive, then we infer that electrostatic complementarity is enhanced by the nullification of 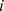, implying that 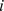 is an electrostatic hindrance to binding affinity.

We may observe that nullification of two amino acids 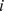 and 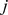 independently result in significant changes to electrostatic complementarity. In such cases, the degree to which electrostatic complementarity is altered by 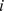 relative to 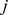 is insufficient to indicate their relative influence on affinity. We cannot draw this inference because other biophysical phenomena may unequally influence electrostatic complementarity for 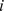 and 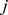, making their relative influence incomparable. We may also observe that an amino acid 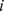 is part of an intramolecular salt bridge and that nullification of 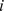 results in a significant change in electrostatic complementarity. In this case, we infer that 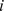 is part of a salt bridge nearby the interface and that mutating 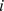 would result in a destabilization of the protein at the interface and a reduction in binding affinity.

Finally, we define two conservative prediction thresholds to predict electrostatically influential amino acids in protein-protein interactions. Given a complex 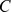 to be evaluated at isopotential threshold 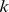, we find the amino acid 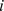 such that 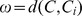 is minimized and the amino acid 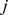 such that 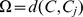 is maximized. Amino acids 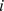 and 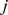 represent the amino acids that most increase and most decrease electrostatic complementarity in 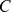 upon nullification. We define the upper prediction threshold 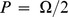. If the nullification of an amino acid 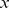 increases electrostatic complementarity beyond 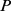, then we predict that it reduces the electrostatic complementarity of the complex enough to reduce affinity. We also define a lower prediction threshold 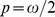. If the nullification of an amino acid 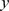 decreases electrostatic complementarity below 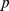, we predict that this amino acid contributes to electrostatic complementarity of the complex enough to enhance affinity. In the case where amino acids at the interface do not act to increase electrostatic complementarity, we only apply the upper prediction threshold if fewer than 10% of the amino acids in the protein cause electrostatic complementarity to surpass 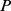. We apply the same requirement to amino acids falling below 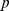.

### 2.5 Clustering cavity fields based on volumetric similarity

Cavity fields based on a given family of proteins were clustered based on the Jaccard distance 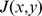.
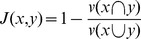
where 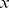 and 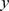 are cavity fields, and 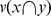 and 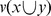 are the volume of the CSG intersection and CSG union of 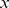 and 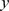, respectively. By normalizing the volume of the intersection by the volume of the union, the Jaccard distance permits cavity fields to be compared without bias relating to total volume. Cavity fields that have a low Jaccard distance have greater volumetric similarity than cavity fields with higher Jaccard distances. Using the neighbor program from Phylip [79], we summarized the pattern of volumetric similarities and variations between all pairs with UPGMA clustering (unweighted pair group method with arithmetic mean).

### 2.6 Clustering other measures of protein similarity

Members of a given family of proteins were also clustered based on amino acid sequence alignments and backbone structure alignments. ClustalW 2.0.7 was used to compute multiple sequence alignments. The resulting alignments were passed to the protpars tool from Phylip [79], to generate a maximum parsimony clustering of the protein sequences. Ska [80] was used to compute backbone structure alignments, which we used to generate a pairwise superposition of every structure onto a selected individual. The root mean squared distance (RMSD) between aligned alpha carbons was clustered via UPGMA, using the neighbor tool from Phylip [79]. Finally, Clustal Omega [81] was used to compute multiple sequence alignments and generate a neighbor joining tree.

### 2.7 Data set construction

Because VASP-E is designed to identify electrostatic influences on specificity, we validate it using families of proteins for which the mechanisms that achieve specificity are well understood and fundamentally electrostatic. The serine protease and cysteine protease superfamilies were selected for validating that VASP-E finds amino acids that influence protein-ligand binding specificity because many mutational studies confirm the role of specific residues in achieving specificity. The same studies permit the validation of VASP-E as a method for clustering proteins based on ligand binding preferences.

The protein data bank (PDB) [73] contains the structures of 681 serine proteases from the trypsin and chymotrypsin subfamilies, and 859 cysteine proteases from the cathepsin B, cathepsin L, and papain subfamilies. From each subfamily, we first removed mutants and functionally undocumented structures. Then we removed structures with greater than 90% sequence identity, creating a nonredundant subset of 12 serine proteases and 4 cysteine proteases. Filtering in this order maximized the number of diverse representative structures. Serine proteases averaged 51% sequence identity and cysteine proteases averaged 40% sequence identity.

We used ska [80] to structurally align the serine proteases to bovine chymotrypsin (pdb: 8gch) and the cysteine proteases to papaya papain (pdb: 1pad). Chymotrypsin and papain were selected because they are in complex with a peptide substrate. Using a method described earlier [37], substrate residues in the S1 subsite of the serine proteases and the S2 subsite of the cysteine proteases were used to generate a solid representation of the binding cavity in all structures. The binding cavity representation and the electrostatic field in each structure was then used to create cavity fields with the method in Section 2.2.

**Figure 5:**
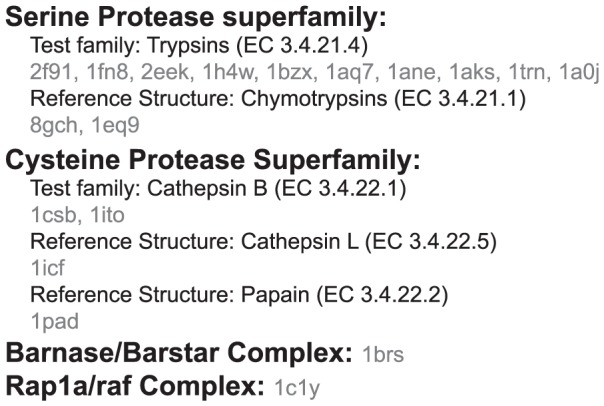
PDB codes of structures used.

We demonstrate the comparison of interface fields on two protein complexes: barnase-barstar (pdb: 1brs) and rap1A-RAF (pdb: 1c1y). We selected these complexes because electrostatic potential is known to affect their binding preferences and because detailed experimental studies have established how binding preferences are affected by mutations on both sides of the interface. These studies create a well-defined gold standard for evaluating how accurately VASP-E can predict amino acids that alter binding preferences. The data set is summarized in Fig. 5.

### 2.8 Implementation details and performance

VASP-E was developed in ansi C/C++ using gcc (the Gnu Compiler Collection) version 4.4.7, on 64 bit linux-based computing platforms. Experimentation was performed on Corona, a cluster at Lehigh University with 1056 Opteron cores (model 6128) running at 2.0 Ghz. Each compute node on corona had 16 cores with access to either 2 or 4 GB of random access memory (RAM) per core. VASP-E is a single-threaded process that runs on one core and approximately 1 GB of random access memory. All experimentation was conducted at .5 Å resolution, which permitted accurate results and practical runtimes.

Visualization for some figures was performed with SURFview, a tool written using the OpenGL library and running on Intel Core i7 and Nvidia Geforce GTX 660 chipsets, in Microsoft Windows 7. Trees representing clusterings were visualized using Newick Utilities [82].

The performance of VASP-E depends on the volume and resolution of the molecular surfaces or electrostatic isopotentials analyzed. On our dataset, generating solid isopotentials for entire proteins required approximately 9.5 seconds on average, to process an average of 1,337,083 lattice cubes. Comparing cavity fields required 1.06 seconds on average, to process an average of 41,984 cubes via CSG, while interface fields from two complexes required 23.4 seconds on average, to process an average of 729,321 cubes.

The website http://www.cse.lehigh.edu/~chen/software.htm hosts the software and primary data associated with this paper for public download.

## Results

### 3.9 Serine proteases

Serine proteases exhibit affinity for amino acids at specificity subsites called S4, S3, …, S1, S1′, …, S3′, S4′ [83]. Each subsite recognizes substrate residues P4, P3, …, P1, P1′, …, P3′, P4′, enabling the protease to selectively cleave the peptide bond between P1 and P1′. Trypsins are digestive serine proteases that narrowly prefer positively charged amino acids [84] at 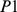. Their selectivity is assisted by the strongly negative electrostatic character of S1. In contrast, chymotrypsins hydrolyze peptide bonds following large hydrophobic amino acids [85] and exhibit considerably less electrostatic potential at their S1 subsite.

**Figure 6:**
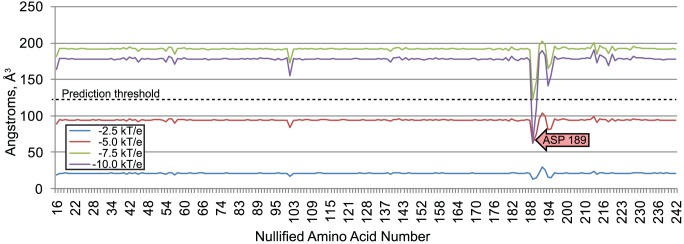
Average volume differences between chymotrypsin and trypsin cavity fields with nullified amino acids. The red arrow indicates a trypsin residue associated with increased electrostatic similarity (downward spikes) when it is nullified. The dashed line represents the average prediction threshold between chymotrypsin and trypsin cavity fields.

Using VASP-E, we identified amino acids that create electrostatic differences between trypsins and chymotrypsins at S1. Fig. 6 reports the average volumetric difference between cavity fields from all trypsins in our dataset and the cavity field of bovine chymotrypsin (pdb: 8gch), where each trypsin residue has been nullified individually. Volume differences were computed for cavity fields generated at −2.5, −5.0, −7.5, and −10.0 kT/e. Volumetric differences between nullified trypsin and chymotrypsin cavity fields varied most at −10 kT/e, so a prediction threshold was computed for differences at this level. The average volumetric difference between trypsin and chymotrypsin cavity fields remained nearly constant for almost all residue nullifications and all four thresholds. Nullifying almost all trypsin residues does not make the very different electrostatic environments of the trypsin and chymotrypsin S1 pockets more similar.

**Figure 7:**
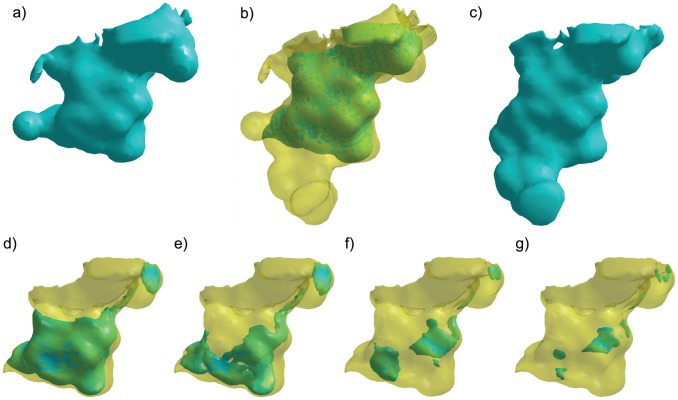
A visual examination of the nullification of aspartate 189 of trypsin. a) S1 cavity of atlantic salmon trypsin (pdb: 1a0j) shown in teal. b) Intersection region (teal) of S1 cavities from trypsin and chymotrypsin (transparent yellow). c) S1 cavity of bovine chymotrypsin (pdb: 8gch) shown in teal. Inset figs. d-g illustrate cavity fields, all with potential less than −10 kT/e (teal), inside the intersection region (transparent yellow). d) The wild type trypsin cavity field occupies 152 Å^3^. e) The trypsin cavity field with D189 nullified (32 Å^3^). f) The wild type chymotrypsin cavity field (9 Å^3^). g) The chymotrypsin cavity field with D189 nullified (2 Å^3^).

One notable exception stands out. Nullifying aspartate 189 in all trypsins results in a large reduction in the average electrostatic difference with chymotrypsin at all potential thresholds, suggesting that the presence of aspartate 189 makes their S1 pockets electrostatically different. Fig. 7 illustrates the effect that nullifying aspartate 189 has on the electrostatic difference between chymotrypsin and trypsin, using bovine chymotrypsin and atlantic salmon trypsin as examples. VASP-E examines only the volumetric intersection of their S1 cavities, where the binding cavities have no steric differences (Fig. 7b). In unmodified trypsin (Fig. 7d), the intersection region exhibits a 152 Å^3^ region with electrostatic potential less than or equal to −10 kT/e. Once D189 is nullified, the region with potential less than or equal to −10 kT/e drops to 32 Å^3^ (Fig. 7e). In comparison, regions of negative electrostatic potential in chymotrypsin, where the S1 cavity overlaps with that of trypsin, is small and remains small when S189 is nullified (Fig. 7f,g). Similar effects were observed with other trypsins. These indications predict experimentally established observations that the negatively charged aspartate 189, at the bottom of the S1 pocket, creates the specificity of trypsin for positively charged amino acids [48], [86].

**Figure 8:**
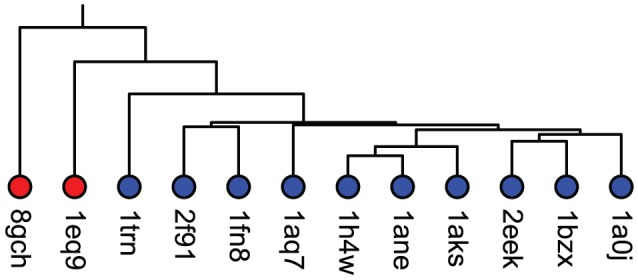
Patterns of electrostatic similarity in the S1 specificity pockets of trypsins and chymotrypsins, relative to P1 binding preferences. The color coding, which is independent of tree topology, indicates the types of P1 residue preferred by each protein. Trypsins (blue) prefer basic amino acids and chymotrypsins (red) prefer large hydrophobic amino acids. The topology of the tree reflects patterns of similarity measured with the Jaccard distance. Proteins on adjacent branches have greater similarity than proteins on different subtrees. The topological separation of the chymotrypsins from the trypsins indicates that similarities and differences in the electrostatic character of S1 subsites, which create the differences in their binding preferences, were detected and correctly classified by VASP-E, using the Jaccard distance.

Fig. 8 illustrates a UPGMA clustering of cavity fields from trypsin and chymotrypsin S1 cavities, generated at −10 kT/e. The topology of the tree, which reflects electrostatic similarities and differences measured with the Jaccard distance, correctly separated the chymotrypsins as outliers from the trypsins. This result indicates that the electrostatic characteristics measured by VASP-E correlate with similarities and differences in serine protease binding preferences.

Clusterings based on cavity fields generated at −2.5, −5.0, −7.5, or −10.0 kT/e (Fig. S1) illustrate that the classification is correct for a range of isopotential thresholds, though they becomes less accurate as thresholds approach neutral charges. Also, relative to comparisons of protein sequences and structures, separated trypsins and chymotrypsins less well (Fig. S2).

### 3.10 Cysteine proteases

Cathepsin B is involved in the onset of pancreatitis [87] and the malignant progress of tumors [88]. Following the same subsite/substrate numbering scheme as serine proteases, Cathepsin B cleaves a peptide bond following two positively charged amino acids that bind in its S1 and S2 subsites [89]. The S2 subsite exhibits a strong negative potential that enables the recognition of positively charged side chains. In contrast, cathepsin L and papain prefer bulky hydrophobic amino acids at 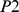 [90], [91], and both exhibit an uncharged S2 subsite [91].

**Figure 9:**
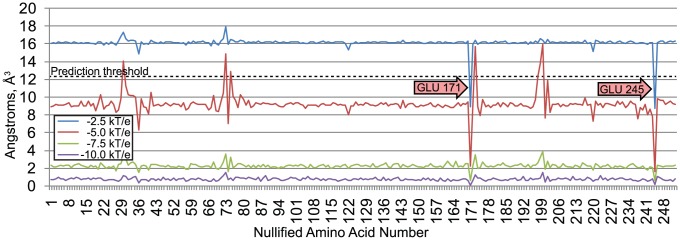
Average volume differences between cathepsin L and cathepsin B cavity fields with nullified amino acids. The red arrows indicate amino acids in cathepsin B associated with increased electrostatic similarity (downward spikes) to cathepsin L, when they are nullified.

We used VASP-E to identify amino acids that create electrostatic differences between cathepsin B and cathepsin L. Fig. 9 illustrates the average volumetric difference between cavity fields representing S2 in cathepsin B and human cathepsin L (pdb: 1icf) generated at −2.5, −5.0, −7.5, and −10.0 kT/e. Volumetric differences between cavity fields with different nullified amino acids were greatest at −2.5 kT/e, so a prediction threshold was computed for differences at this level. The average volumetric difference was nearly constant for almost all residue nullifications. Insignificant fluctuations in the volumetric difference were approximately the same magnitude as in serine proteases.

The nullification of two amino acids, glutamic acid 171 or glutamic acid 245, reduced electrostatic differences between cathepsin B and cathepsin L beyond the prediction threshold. This observation suggests that both amino acids create electrostatic differences between the S2 subsites of cathepsin B and L. Indeed, glutamic acid 245 has been shown to cause Cathepsin B to bind arginine residues at the S2 cavity [90], while cathepsin L prefers phenylalanines. In glutamic acid 171, one of the carboxylate oxygens is involved in a hydrogen bond and the other is free to form other interactions in the S2 pocket. Such interactions have been observed with positively charged inhibitors [92], [93], again in contrast with cathepsin L.

**Figure 10:**
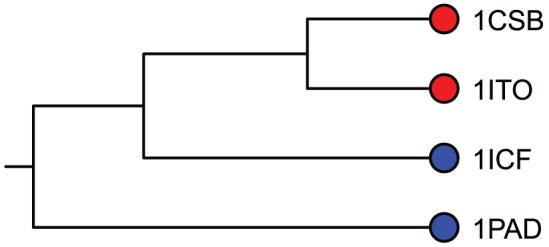
Patterns of electrostatic similarity in the S2 specificity pockets of cathepsin B, cathepsin L, and papain. The color coding, which is independent of tree topology, indicates the types of P2 residue preferred by each protein. Cathepsin B's (red) prefer basic amino acids and cathepsin L and papain (blue) prefer large hydrophobic amino acids. The topology of the tree reflects patterns of similarity measured with different comparison algorithms. Proteins on adjacent branches have greater similarity than proteins on different subtrees. The topological separation of the cathepsin B's from cathepsin L and papain indicates that similarities and differences in the electrostatic character of S2 subsites, which create the differences in their binding preferences, were detected and correctly classified by VASP-E, using the Jaccard distance.

Fig. 10 plots a UPGMA clustering of cavity fields based on S2 subsites from cysteine proteases in our data set. Cavity fields were generated at −2.5 kT/e. The topology of the tree describes electrostatic similarities and differences measured with the Jaccard distance. It is apparent that the tree structure clusters cathepsin B cavities, setting them apart from those of cathepsin L and papain, which have different binding preferences. Cavity fields produced at −2.5, −5.0, −7.5, and −10.0 kT/e, shown in Fig. S3, cluster in a similar manner. This pattern of separations demonstrates that VASP-E is correctly identifying electrostatic conservations and variations that correlate to binding preferences in the S2 subsite. Global sequence and structure alignments separated the cysteine proteases as well as the Jaccard distance clustering (Fig. S4).

### 3.11 Barnase-barstar

Barnase is an guanine-preferring endo-ribonuclease expressed by Bacillus amyloliquefaciens [94] whose activity, without inhibition by barstar, can be lethal to the cell [95]. Barstar inhibits barnase by forming an extremely tight complex with close steric and electrostatic complementarity at many amino acids across the binding site [96]. We used VASP-E to identify mutations that enhance or diminish electrostatic complementarity.

#### 3.11.1 Nullifications of barnase amino acids

**Figure 11:**
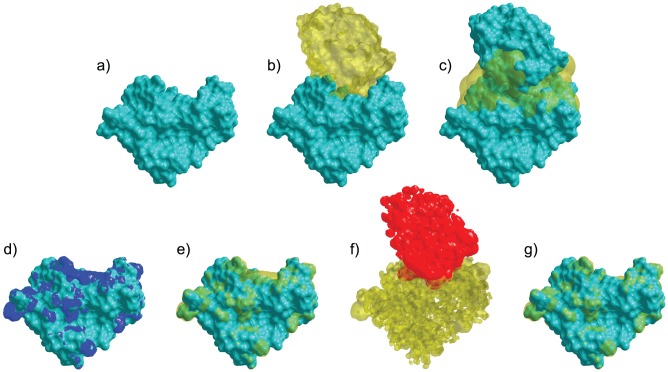
Volume differences between interface fields of wildtype barnase/barstar and a barnase/barstar complex with electrostatic nullifications in the barnase residues. The red arrows indicate amino acids in barnase that are associated with decreased electrostatic complementarity with barstar, when they are nullified. Blue arrows indicate amino acids associated with increased electrostatic complementarity, when they are nullified. Green arrows indicate amino acids below the prediction threshold that are known to influence specificity.

Fig. 11 illustrates comparisons of wildtype and modified barnase-barstar interface fields, where nullifications were performed on Barnase. The magnitude of volume changes observed were much larger than in the cavity fields examined earlier because the volume of the interface is much larger than the cavities. We evaluated the impact of nullifying individual amino acids with values of 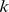 equal to 1.0, 3.0, 5.0, 7.0, and 9.0 kT/e. Differences in electrostatic complementarity caused by nullifying some amino acids were greatest at 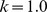 kT/e, so this value of 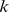 was used to set upper and lower prediction thresholds. Nullification of most barnase amino acids resulted in small changes in electrostatic complementarity below both prediction thresholds. However, nullification of a few amino acids created very large increases and decreases in complementarity between wildtype and modified interface fields.

For four barnase residues, K27, R59, R83, and R87, nullification significantly reduced electrostatic complementarity, predicting correctly that mutations abrogating net charge at these positions could reduce affinity. These predictions are consistent with experimental observations established earlier: K27A decreases association rates by a factor of 7 to 10 times [97]–[99]. R59A reduces association rates by a factor of 7 to 10 times [98], [99]. R83A decreases association rates by 4 to 6 fold [95], [97], [99]. R87A decreases association rates by 2 to 3 fold [97]–[99].

Nullification of barnase residues 54 and 73 significantly increased electrostatic complementarity, correctly predicting that substituting these amino acids with alanine should increase affinity. Predictions for D54 and E73 reproduced established observations: Substituted individually, D54A and E73A increase association rates by 2 to 4 fold [98], [99]. Also, D75 is involved in an intramolecular salt bridge, and is thus predicted to reduce the stability of barnase and it's ability to form a complex with barstar. This prediction is correct; the substitution of D75 with asparagine, a nearly isosteric but uncharged analogue of aspartate, is known to diminish complex stability by 4.80 kcal/mol [100].

Three known influences on affinity fell below our prediction threshold. Nullifying residues 39 and 102 reduced electrostatic complementarity, but not significantly enough to achieve our prediction threshold. The mutation K39A is known to reduce affinity [95], and the mutation H102A reduces association rates less than 2 fold [97]–[99]. Also, it is known that replacing glutamic acid 60 with alanine is known to increase association rates by 2 to 4 fold [98], [99], but nullifying glutamic acid 60 did not generate a large increase in electrostatic complementarity. While VASP-E made no incorrect predictions, the application of a conservative prediction threshold caused some influential amino acids to be missed.

**Figure 12:**
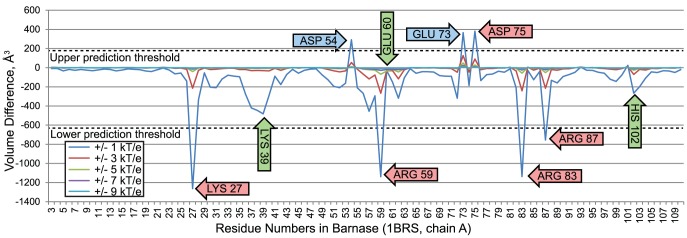
A visual examination of the nullification of lysine 27 in barnase. a) The molecular surface of Bacillus amyloliquefaciens barnase (teal). b) The molecular surface of Bacillus amyloliquefaciens barstar (transparent yellow) and barnase (teal). c) The interface region (transparent yellow) between barnase and barstar (teal). d) Electrostatic isopotential at +3 kT/e (blue) near barnase (teal). e) The same isopotential shown in transparent yellow. f) The electrostatic isopotential at −3 kT/e near barstar (red) and it's overlap with the electrostatic isopotential at +3 kT/e near barnase (transparent yellow). g) Electrostatic isopotential at +3 kT/e (blue) near barnase (teal), where Lysine 27 is nullified.

Nullifications of influential amino acids identified by VASP-E create changes in electrostatic complementarity that can be localized to specific regions. For example, Fig. 12 illustrates the effect of nullifying lysine 27 in barnase. In the interface region, lysine 27 is responsible for a large positively charged region of electrostatic potential that extends outwards towards barstar (Fig. 12e). This region overlaps considerably with a negative isopotential from barstar (Fig. 12f). When lysine 27 is nullified, the positively charged isopotential on barnase collapses (Fig. 12g), and electrostatic complementarity is substantially reduced. This ability to identify spatial regions of electrostatic complementarity, and thus provide insights into the mechanisms that control specificity, is unique to VASP-E.

##### 3.11.2 Nullifications of barstar amino acids

**Figure 13:**
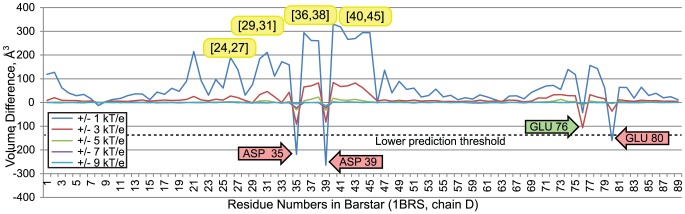
Volume differences between interface fields of wildtype barnase/barstar and a barnase/barstar complex with electrostatic nullifications in the barstar residues. The red arrows indicate amino acids in barstar that are associated with decreased electrostatic complementarity with barstar, when they are nullified. Numbers in yellow ovals indicate inclusive intervals of amino acids where electrostatic focusing enhances the volume of the electrostatic potential inside the barnase/barstar interface. The green arrow indicates an amino acid below the prediction threshold that is known to influence specificity.

Fig. 13 plots comparisons of wildtype and modified Barnase-Barstar interface fields, where nullifications were performed on Barstar. Because Barstar is interacting with Barnase, the same calibration threshold, 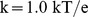, was used. Also, because more than 10% of amino acids in barstar are above the threshold, an upper prediction threshold was not used, suggesting that there are no outliers on the positive end. Nullifications of several amino acids created distinctive differences between wildtype and modified interface fields.

Nullifying three barstar residues, 35, 39 and 80 reduced electrostatic complementarity. These observations correctly predict experimental observations that these amino acids are crucial for affinity between barnase and barstar, and that diminishing their electrostatic contribution interferes with binding: Charge reversal mutations individually converting aspartate 35 and 39 to lysine were shown to halt the inhibition of barnase by barstar [99], [101]. Mutation of glutamic acid 80 to alanine reduces the binding energy by .5 kcal/mol and increases the dissociation constant by 2.5 fold relative to the wildtype complex.

The nullification of glutamic acid 76 insufficiently reduced electrostatic complementarity to be associated with a prediction. Nonetheless, the mutation of E76 to alanine was shown to reduce the binding energy by 1.6 kcal/mol and increases the dissociation constant by 10 fold relative to the wildtype complex [99]. As was the case with barnase, VASP-E made no incorrect predictions but the application of a conservative prediction threshold caused some influential amino acids to be missed.

Nullifying the uncharged interfacial amino acids 29–31, 36–38, and 40–46 generated increases in electrostatic complementarity via electrostatic focusing. This enhancement creates isopotentials with larger volume, especially when the isopotentials are generated at low absolute thresholds (e.g.+/− 1 kT/e). Since these amino acids are uncharged, their nullification enlarges the isopotentials of nearby charged amino acids D35 and D39.

### 3.12 Rap1a-Raf

Ras is a master regulator that transmits a wide range of signals via protein-protein interactions. Downstream, its effectors are involved in many crucial systems, including cell cycle progression, cell division, apoptosis, lipid metabolism, DNA synthesis, and cytoskeletal organization [102]–[104]. While the structure of ras in complex with these effectors is unknown, rap1a, a homolog of ras (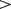
50% sequence identity), can serve as a substitute. Rap1a has an essentially identical binding interface and binds competitively with the same downstream effectors [105], such as raf, an oncogene involved in ERK 1/2 signaling [106]. Here, we use VASP-E to examine the effect of charge nullification on the rap1a-raf interface to make predictions on the effect of mutation on ras-raf binding.

#### 3.12.1 Nullifications of Rap1A amino acids

**Figure 14:**
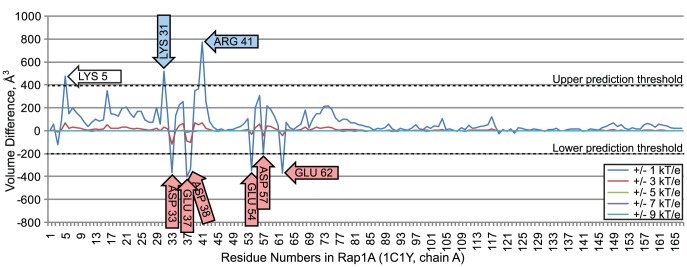
Volume differences between the interface fields of a wildtype rap1a/raf complex and a rap1a/raf complex with electrostatic nullifications in rap1a residues. The red arrows indicate amino acids in rap1a that are associated with decreased electrostatic complementarity with barstar when they are nullified. Blue arrows indicate amino acids associated with increased electrostatic complementarity, when they are nullified. The white arrow indicates an open prediction.

Fig. 14 plots comparisons of wildtype and modified Rap1A-Raf interface fields, where nullifications were performed on Rap1a. We evaluated the impact of nullifying individual amino acids with values of 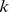 equal to 1.0, 3.0, 5.0, 7.0, and 9.0 kT/e. Differences in electrostatic complementarity caused by nullification were greatest at 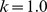 kT/e, so this value of 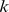 was used to set upper and lower prediction thresholds. Nullification of most barnase amino acids resulted in small changes in electrostatic complementarity below both prediction thresholds. However, nullification of several amino acids created very large increases and decreases in complementarity between wildtype and modified interface fields.

Nullification of six rap1a residues, 33, 37, 38, 54, 57 and 62 reduced electrostatic complementarity beyond the lower prediction threshold, suggesting that loss of charge mutations would reduce complex affinity. These predictions were consistent with established experimental observations: Substituting aspartate 33 for alanine in rap1a results in a binding energy reduction of 1.2 kcal/mol [107]. Glutamic acid 37, in both rap1a and ras, forms hydrogen bonds with with R59 and R67 in raf [105]. Substituting E37 with glycine would break these bonds, and in ras-raf, E37G inhibits the formation of the complex [108]. Substituting aspartate 38 for alanine in ras, eliminating its contribution to electrostatic complementarity and removing a hydrogen bond, reduces its rate of association with raf by 72 fold [107], [109]. Glutamic acid 54 forms a hydrogen bond with arginine 67 of raf. Mutations of R67 that break this bond reduce the rate of association by 12 fold [108], [110]. Substituting E54 with alanine would break the same bond and likely achieve a similar effect. Substituting aspartate 57 for alanine in Ha-Ras causes a total loss of affinity to raf [111]. Finally, glutamic acid 62 is a conserved amino acid that radically affects binding in a range of RAS homologs when mutated [112].

Nullification of two rap1a residues, 31 and 41, increased electrostatic complementarity beyond the upper prediction threshold, suggesting that mutations removing their net charge should also increase affinity. Established results confirm these observations: Charge reversal of lysine 31 to glutamic acid is known to create a 30 fold increase in affinity [110]. In Ha-ras, a substitution of arginine 41 to alanine is known to increase affinity by 11.8 fold [111], [113].

Finally, VASP-E predicted that the nullification of lysine 5 could result in an increase in binding affinity. This observation suggests that lysine 5 may normally reduce affinity. However, to our knowledge, no current experimental results that establish this claim, and hence we leave it as an open prediction.

#### 3.12.2 Nullifications of Raf amino acids

**Figure 15:**
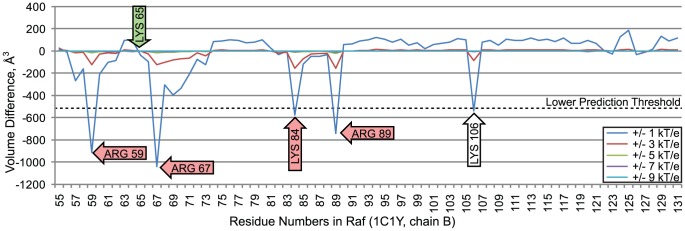
Volume differences between the interface fields of a wildtype rap1a/raf complex and a rap1a/raf complex with electrostatic nullifications in raf residues. The red arrows indicate amino acids in rap1a that are associated with decreased electrostatic complementarity with barstar when they are nullified. The green arrow indicates an amino acid below the prediction threshold that is known to influence specificity. The white arrow indicates an open prediction.

Fig. 15 plots comparisons of wildtype and modified Rap1A-Raf interface fields, where nullifications were performed on residues in raf. Because Raf is interacting with Rap1a, the same calibration threshold, 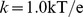, was used. Also, because more than 10% of amino acids in Raf are above the threshold, an upper prediction threshold was not used, suggesting that there are no outliers on the positive end. Nullifications of several amino acids created distinctive differences between wildtype and modified interface fields.

Nullification of four residues in raf, 59, 67, 84, and 89 reduced electrostatic complementarity below the lower prediction threshold and correctly predicted experimentally established substitutions that correspond to reductions in affinity. Substituting arginine 59 with alanine is known to reduce the rate of association by 25 fold [108], [110]. Substituting arginine 67 with alanine is known to reduce the rate of association by 12 fold [108], [110]. Also, both arginine 59 and 67 form hydrogen bonds with glutamic acid 37 in rap1a. Loss of these hydrogen bonds inhibits complex formation [108]. Substitution of lysine 84 with alanine produces a 9.4 fold reduction in the association rate [110], and substitution of arginine 89 with leucine inhibits complex formation [105].

Nullification of lysine 65 did not reduce electrostatic complementarity below the lower threshold. While lysine 65 was therefore not predicted to have a significant electrostatic influence on specificity, the mutation K65A is known to reduce the rate of association by 4.5 fold [110]. While VASP-E made no incorrect predictions, the conservative prediction threshold caused K65 to be overlooked.

### 3.13 Analysis of prediction performance on individual amino acids

By collecting the predictions made on our dataset, we can measure the prediction performance of VASP-E. We begin by counting true positives (TPs), false positives (FPs), true negatives (TNs), and false negatives (FNs). TPs are defined as amino acids that are both predicted by VASP-E to have an influence on specificity and also published in experimental findings to have such an influence. The predictions detailed earlier in this section cite these findings as specific validation for the predictions made with VASP-E. FPs are amino acids that are both predicted by VASP-E to have an influence on specificity and are documented in the literature to not have an effect on specificity. TNs are amino acids that are predicted to not have an influence on specificity that are also documented in the literature to not have an effect on specificity. FNs are amino acids predicted to not have an influence on specificity but are established in the literature as having a role in specificity. Finally, we VASP-E made two predictions that were neither confirmed nor denied in the literature. We leave these two observations as open predictions and do not include them in our evaluation of prediction performance.

**Figure 16:**
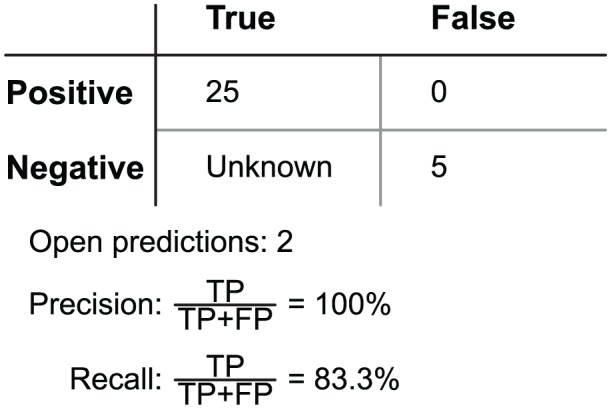
Precision and recall performance of VASP-E.

Of these statistics, TNs cannot be fully counted because no studies categorically classify the role of every amino acid in specificity, including those that are distant from the binding site. For this reason, we describe the number of true negatives as unknown. Nonetheless, we do not require TNs in order to compute *precision* and *recall*, two fundamental statistics used to evaluate the accuracy of a predictor. Precision is the fraction of predictions that are verified in experimental studies and recall is the fraction of verified experimental results that are correctly predicted. Using our conservatively defined prediction thresholds, every prediction made with VASP-E was verified, giving perfect precision, and most verified results were correctly predicted, giving strong recall. Precision and recall are reported together in Fig. 16.

## Discussion

We have presented VASP-E, a new program for the comparison of electrostatic isopotentials. To our knowledge, VASP-E is the first program capable of comparing isopotentials using CSG, enabling a new unified approach to the characterization of protein-ligand and protein-protein binding specificity. In an application to the serine and cysteine proteases, we demonstrate that VASP-E is capable of reproducing known ligand binding preferences and of detecting differences in electrostatic potential among proteins that, based on global sequence and structure similarity, might have been expected to be similar. Subtle differences like these, which can arise from variations in single amino acids, can still be detected by VASP-E because they are reflected in differently shaped isopotentials.

Central to our approach is a novel solid representation of electrostatic isopotentials that can also represent regions within molecular surfaces. This seamless integration of two nearly orthogonal aspects of protein structure enables analytical capabilities that were not possible before. One capability is the identification of amino acids that create differences in electrostatic isopotentials at binding cavities. Using the molecular surface to exclude electrostatic variations outside the binding cavity, we identified three amino acids in trypsin and cathepsin B that create electrostatic differences in binding specificity. These predictions correctly reflected experimentally established observations regarding their electrostatic influence. VASP-E also finds amino acids that change electrostatic complementarity in protein-protein interfaces. In an analysis of the barnase-barstar and rap1a/raf complexes, VASP-E predicted 22 amino acids that either increase or decrease affinity upon mutation, all in agreement with established experimental results. Solid representations enable a deconstructive analysis of electrostatic fields that permits the discovery of individual residues that influence binding preferences in protein-ligand and protein-protein binding sites.

As the first approach to the comparison of electrostatic isopotentials with CSG, VASP-E exhibits novel potential for useful experimental applications. In experimental settings, identifying mutants that may alter binding specificity can be a time consuming and expensive effort with many possible mutants to consider. VASP-E identifies amino acids that might play a role in specificity, and, in addition, it suggests a biophysical mechanism for that amino acid: It may increase or decrease electrostatic complementarity. This additional information, beyond simply identifying an important amino acid, provides utility beyond the identification of important amino acids because it suggests how that amino acid might be tested, such as by mutation to an uncharged or oppositely charged residue. When comparing protein-ligand binding cavities, pointing out amino acids that create electrostatic differences can inform experimental design.

VASP-E has the potential to serve broad applications. For example, identifying groups of amino acids that work together to achieve specificity can be an especially difficult problem, because of the combinatorial space of variants that must be considered. Nullification, as applied to individual amino acids in this paper, could be exhaustively applied to many combinations of residues to assist in experimental design. Given the rapid performance of VASP-E and the expanding availability of parallel computing, examining combinations of influential amino acids would also be very practical. Furthermore, the analysis of influential amino acids at protein-protein interfaces is not limited to dimers; the approach described here could be logically extended to higher order interactions. For such applications, interfaces between specific chains could be considered individually or in groups, to reflect the order in which the complex associates. Finally, while VASP-E is designed to identify subtle variations among highly similar proteins, VASP-E could in principle be used to analyze electrostatic similarities and differences among binding sites from very different proteins, as long as structural alignments could be correctly generated and binding cavities can be properly defined. These diverse applications suggest that the integrated representation and comparison of structure and electrostatics may offer an important new tool in the study of drug resistance and algorithms for specificity annotation.

## Supporting Information

Figure S1 Patterns of electrostatic similarity in the S1 specificity pockets of trypsins and chymotrypsins, relative to P1 binding preference. The color coding in all trees, which is independent of tree topology, indicates the types of P1 residue preferred by each protein. Trypsins (blue) prefer basic amino acids and chymotrypsins prefer large hydrophobic amino acids (red). The topology of each tree reflects patterns of similarity measured with the Jaccard distance on cavity fields generated at different isopotential thresholds. In each tree, proteins on adjacent branches have greater similarity than proteins on different subtrees. The topology of the trees reflect UPGMA clustering of serine protease cavity fields generated at (a) 2.5 kT/e, (b) 5.0 kT/e, (c) 7.5 kT/e, (d) and 10.0 kT/e.
(EPS)

Figure S2 Patterns of similarity and variation in the sequence, backbone structure, and cavity fields of trypsins and chymotrypsin, relative to P1 binding preference. The color coding in all trees, which is independent of tree topology, indicates the types of P1 residue preferred by each protein. Trypsins (blue) prefer basic amino acids and chymotrypsins prefer large hydrophobic amino acids (red). The topology of each tree reflects patterns of similarity measured with different comparison algorithms. In each tree, proteins on adjacent branches have greater similarity than proteins on different subtrees. The topology of tree (a) reflects sequence similarity measured with Clustalw 2.0.7, the topology of (b) reflects backbone structure similarity measured with ska, the topology of (c) reflects cavity field similarity measured with the Jaccard distance, and the topology of (d) reflects sequence similarity as measured with clustal omega. Jaccard similarity positions serine proteases with similar P1 binding preferences more closely than the other similarity measures do.
(EPS)

Figure S3 Patterns of electrostatic similarity in the S2 specificity pockets of cathepsin B, cathepsin L, and papain, relative to P2 binding preference. The color coding in all trees, which is independent of tree topology, indicates the types of P2 residue preferred by each protein. Cathepsin B's (red) prefer basic amino acids and cathepsin L and papain (blue) prefer large hydrophobic amino acids. The topology of each tree reflects patterns of similarity measured with the Jaccard distance on cavity fields generated at different isopotential thresholds. In each tree, proteins on adjacent branches have greater similarity than proteins on different subtrees. The topology of the trees reflect UPGMA clustering of cysteine protease cavity fields generated at (a) 2.5 kT/e, (b) 5.0 kT/e, (c) 7.5 kT/e, (d) and 10.0 kT/e.
(EPS)

Figure S4 Patterns of similarity and variation in the sequence, backbone structure, and cavity fields of cysteine proteases, relative to P2 binding preference. The color coding in all trees, which is independent of tree topology, indicates the types of P2 residue preferred by each protein. Cathepsin B's (red) prefer basic amino acids and cathepsin L and papain (blue) prefer large hydrophobic amino acids. The topology of each tree reflects patterns of similarity measured with different comparison algorithms. In each tree, proteins on adjacent branches have greater similarity than proteins on different subtrees. The topology of tree (a) reflects sequence similarity measured with Clustalw 2.0.7, the topology of (b) reflects backbone structure similarity measured with ska, the topology of (c) reflects cavity field similarity measured with the Jaccard distance, and the topology of (d) reflects sequence similarity as measured with clustal omega. Jaccard similarity positions cysteine proteases with similar P2 binding preferences in a manner similar to the other similarity measures.
(EPS)

Text S1 Text S1 includes three supplemental notes describing the calibration of the method.
(PDF)
