# Supplementary material for: VASP-E: Specificity Annotation with a Volumetric Analysis of Electrostatic Isopotentials
Source: PLoS Comput Biol. 2014 Aug 28;10(8):e1003792. doi: 10.1371/journal.pcbi.1003792 (PMC4148194; doi:10.1371/journal.pcbi.1003792)
Supplement: Text S1 — includes three supplemental notes describing the calibration of the method. (PDF) [file pcbi.1003792.s005.pdf]

Text S1

Calibration of kT/e thresholds. In this paper, applications of VASP-E to the identification of amino acids that affect protein-protein and protein-ligand binding specificity are described using a threshold of electrostatic potential k. To calibrate k, the user must first determine if they are interested in examining the positive or negative sides of the electrostatic field. This question can be answered based on their general knowledge of protein they are studying: if they believe that positive potentials may influence binding, they should examine positive potentials, and if they believe that negative potentials may influence binding, they should examine negative potentials. Both can also be considered. A visualization of electrostatic potentials, such as that provided by GRASP [12], can provide the necessary insights.

Calibration for ligand binding cavities. Next, VASP-E can loop through a range of electrostatic potentials with the polarity selected by the user, starting from high absolute values, such as ± 10 kT/e and testing intervals of 2 to 4 kT/e towards zero. This approach is not highly sensitive to calibration different values of k, because the influence of specific amino acids on the geometry of the isopotential does not change in a nonmonotonic manner. As a result, amino acids that have a significant influence on the electrostatic field of the binding cavity, such as those associated with spikes shown in Fig. 6 and Fig. 9, remain significant relative to other amino acids, within a wide range of values for k: When k is too large, the volume differences between the cavity fields under comparison approach zero, as can be seen in Fig. 9, and when k is too small, it approaches a capped maximum volume defined by the binding cavity. Looping through the intermediate values within this range in coarse steps, as shown in Fig. 6 and Fig. 9, rapidly reveals a threshold at which influential amino acids are clearly significant relative to others: We select the threshold with the largest spikes. Cavity field clusterings, as shown in Fig. S1 and Fig. S3, should use the same threshold k.

Calibration for protein-protein interfaces. Calibration for identifying influential amino acids at protein-protein interfaces is performed similarly to that of ligand binding cavities. Note first that the analysis of protein-protein interfaces examines isopotentials at both positive k and negative k, and as such selecting the initial polarity is unnecessary. Starting from high absolute values, such as ± 10 kT/e and testing intervals of 2 to 4 kT/e towards zero, k is correctly calibrated again when individual amino acids exhibit large spikes that differ significantly from most amino acids, which, when nullified, will cause interface field differences close to zero. In this situation as well, calibration is insensitive to different values of k, because the influence of specific amino acids on the geometry of the isopotential is monotonic. This monotonic scaling is apparent in Fig. 11, Fig. 13, Fig. 14 and Fig. 15, where spikes at the same amino acid reveal the scaling effect.
